# Combining Hyperspectral Techniques and Genome-Wide Association Studies to Predict Peanut Seed Vigor and Explore Associated Genetic Loci

**DOI:** 10.3390/ijms25158414

**Published:** 2024-08-01

**Authors:** Zhenhui Xiong, Shiyuan Liu, Jiangtao Tan, Zijun Huang, Xi Li, Guidan Zhuang, Zewu Fang, Tingting Chen, Lei Zhang

**Affiliations:** Guangdong Provincial Key Laboratory of Plant Molecular Breeding, College of Agriculture, South China Agricultural University, Guangzhou 510642, China; xzhgreatdd@163.com (Z.X.); 2234272317@scau.edu.cn (S.L.); 20222014016@stu.scau.edu.cn (J.T.); qingnankk@163.com (Z.H.); 15992177933@163.com (X.L.); zgdorange@163.com (G.Z.); f1683365156@outlook.com (Z.F.)

**Keywords:** peanut, seed vigor, hyperspectral techniques, machine-learning, genome-wide association analysis

## Abstract

Seed vigor significantly affects peanut breeding and agricultural yield by influencing seed germination and seedling growth and development. Traditional vigor testing methods are inadequate for modern high-throughput assays. Although hyperspectral technology shows potential for monitoring various crop traits, its application in predicting peanut seed vigor is still limited. This study developed and validated a method that combines hyperspectral technology with genome-wide association studies (GWAS) to achieve high-throughput detection of seed vigor and identify related functional genes. Hyperspectral phenotyping data and physiological indices from different peanut seed populations were used as input data to construct models using machine learning regression algorithms to accurately monitor changes in vigor. Model-predicted phenotypic data from 191 peanut varieties were used in GWAS, gene-based association studies, and haplotype analyses to screen for functional genes. Real-time fluorescence quantitative PCR (qPCR) was used to analyze the expression of functional genes in three high-vigor and three low-vigor germplasms. The results indicated that the random forest and support vector machine models provided effective phenotypic data. We identified *Arahy.VMLN7L* and *Arahy.7XWF6F*, with *Arahy.VMLN7L* negatively regulating seed vigor and *Arahy.7XWF6F* positively regulating it, suggesting distinct regulatory mechanisms. This study confirms that GWAS based on hyperspectral phenotyping reveals genetic relationships in seed vigor levels, offering novel insights and directions for future peanut breeding, accelerating genetic improvements, and boosting agricultural yields. This approach can be extended to monitor and explore germplasms and other key variables in various crops.

## 1. Introduction

Peanuts (*Arachis hypogaea* L.) are a widely cultivated leguminous crop [1], rich in essential nutrients, such as protein, unsaturated fatty acids, and multivitamins [2], offering high nutritional and economic value. According to the Food and Agriculture Organization (FAO), the global peanut harvested area was approximately 30.54 million ha, with a total production exceeding 54 million tons in 2022 (FAOSTAT, accessed on 1 January 2024). The growing population is driving the expansion of urban areas [3], which worsens land degradation and contributes to a decline in arable land [4]. Seeds are fundamental in agricultural production [5] and serve as a delivery system for transferring genetic traits that produce superior phenotypes in the field [6]. Seed vigor is a crucial phenotype that boosts yield by affecting seed characteristics such as seedling emergence, uniformity, and resistance in high-vigor seeds [7,8]. Moreover, genetic factors have a significant effect on regulating seed vigor [9]. Hence, selecting and breeding high-vigor seeds can greatly contribute to agricultural development, and seed vigor testing is an essential prerequisite for these tasks. Conventional vigor testing methods include standard germination tests [10], 2,3,5-triphenyltetrazolium chloride (TTC) staining [11], electrical conductivity measurements [12], and accelerated aging tests [13]. However, these methods are hindered by their time-consuming and burdensome nature and their irreversible seed damage. These limitations make rapid and non-destructive detection difficult, failing to meet the demands of modern agriculture and breeding.

To achieve non-invasive and high-throughput seed vigor detection, spectroscopy technology has made significant contributions and is widely applied to rice [14,15], wheat [16,17], corn [18,19], soybean [20], peanut [21], and other crops. This technique utilizes spectral reflection from molecular transitions to convey internal molecular structures [22], enabling high-throughput monitoring of crop traits, such as water [23], nitrogen [24], oil [25], and chlorophyll content [26]. Additionally, hyperspectral techniques can rapidly determine the reflection and absorption features of a large number of chemical substances in a target plant through reflectance in numerous narrow wavebands [27], which is beneficial for studying complex traits in plants. However, few studies have focused on efficient and non-destructive methods for predicting seed vigor.

Hyperspectral data provide comprehensive spectral information across wide wavelengths but include redundant information and noise, posing challenges for processing. High dimensionality and redundancy lead to monitoring models failing to classify and predict targets accurately owing to interference in the algorithm’s learning process. Principal component analysis (PCA) effectively reduces the dimensionality of spectral data during the initial processing while retaining key information characteristics in the dataset. As computer hardware performance advances and data size increases, machine learning (ML) algorithms can be efficiently used to analyze complex hyperspectral information [28]. Popular algorithms, such as linear regression (Line), support vector machine (SVM), random forest (RF), and random tree (RT), screen relevant features for learning, iteratively enhance them to achieve objectives and proficiently perform prediction or classification tasks. In this study, PCA was employed for dimensionality reduction, and ML algorithms were used to construct regression models.

Genome-wide association studies (GWAS) have been employed to explore potential relationships between genotypes and phenotypes [29,30,31], aiding in identifying genetic variations influencing complex traits. Seed vigor, which comprises numerous physiological traits, is regulated by multiple quantitative loci [32,33]. GWAS are commonly utilized in breeding to explore the genetic relationships between phenotypes and genes. This is evident in numerous studies that have identified candidate genes that enhance seed nutrient composition and size in Medicago truncatula [34], screened quantitative trait loci (QTL), and candidate genes associated with seed vigor in rice [35], as well as identified genes regulating seed size, length, width, and weight traits in peanuts through transgenic experiments [36]. Multiple repeated experiments are conducted to minimize errors in the genome-wide association analysis. However, numerous seed varieties and strains pose challenges for traditional vigor phenotype data collection and GWAS analysis in breeding. Hyperspectral remote sensing technology offers rapid monitoring and acquisition of large amounts of phenotypic data, effectively meeting GWAS data volume requirements while reducing manual sampling errors. This strengthens the correlation between seed phenotypic and genotypic data, enhancing crop improvement efforts.

Therefore, this study aims to investigate an efficient phenotypic monitoring approach using hyperspectral data containing rich target information to rapidly and non-destructively monitor changes in seed vigor. This could facilitate exploring functional genes potentially associated with phenotypic changes. The study seeks to (a) construct a predictive model using ML regression algorithms to identify the predictive phenotypes for peanut seed vigor, and (b) integrate GWAS with hyperspectral data to identify genes potentially related to the peanut seed vigor phenotype. This study is believed to be substantially implicated in rapidly assessing seed vigor, exploring candidate gene loci, and facilitating the breeding of high-vigor peanut seeds in the future.

## 2. Results

### 2.1. Seed Vigor Variation of Peanut Exhibits Diversity

To explore the vigor of peanut seeds from different varieties and treatments, all vigor data (Table 1 and Table 2) showing varied levels of vigor among different seed treatments were recorded. The vigor indicators (GE, GP, and GI) of aging seeds showed a negative correlation with the number of aging days, indicating that seed vigor decreased as the number of aging days increased. The CK (Untreated control group), A3 (Aging 3d), and A6 (Aging 6d) treatments showed no significant differences in the average and maximum values of all indicators. This indicates that in a short period, peanut seeds are less affected by aging mechanisms and can germinate normally. However, from A6 to A9 (Aging 9d), a significant decrease of approximately 50% was observed in the average and standard deviation (SD) of all indicators. This suggests that the aging mechanism in the seed significantly affects the physiological state of the seed over a longer aging period, leading to a reduction in seed vigor. Moreover, this result reveals that the vigor characteristics of peanut seeds have some buffering capacity against the germplasm aging process.

Different peanut varieties expressed diverse vigor levels. Significant changes in the minimum values of each indicator were observed in A3 and A6 compared to CK. This was probably because the overall activity of the germplasm, which already had low vigor, was further reduced by the effects of aging. Additionally, these indicators varied considerably among the varieties subjected to the aging treatments (Table 2). This suggested that aging-affected changes in vigor were related to the characteristics of seed variety. The highest variability across all experiments was demonstrated in A3 and A6 based on the SD, indicating that these aging durations could reveal vigor characteristics across different varieties. From A9 to A18 (Aging 18d), the SD differences were relatively small, indicating a gradual decline in the vigor levels of primary peanut seeds with increasing aging days, tending to converge. In A9, the maximum values were 0.8 for GE, 0.5 for GP, and 11.25 for GI, suggesting the presence of some peanut seeds with high-vigor characteristics. 

Different peanut varieties displayed diverse vigor levels across different treatments. Some varieties showed significant differences in performance based on the SDs of N1 (Natural population 1) and N2 (Natural population 2). Additionally, significant differences were observed in P1, P2, and P3 performance across different aging durations (Table 2). N1 and CK showed similar vigor levels but differed significantly from N2. This may be attributed to two reasons: (a) seeds suffered natural aging between germination tests, and (b) N2 contained more varieties, resulting in consistently high SDs (GE: 0.3563; GP: 0.2732; GI: 4.9333) for the indexes, indicating considerable variation in vigor performance among different varieties.

In summary, the vigor datasets of peanut seeds demonstrated remarkable diversity, which could aid in the iterative learning of algorithms, thereby enhancing the ability of monitoring models to effectively handle various detection targets.

### 2.2. SVM and RF Models Predict Peanut Seed Vigor 

**Figure 1:**
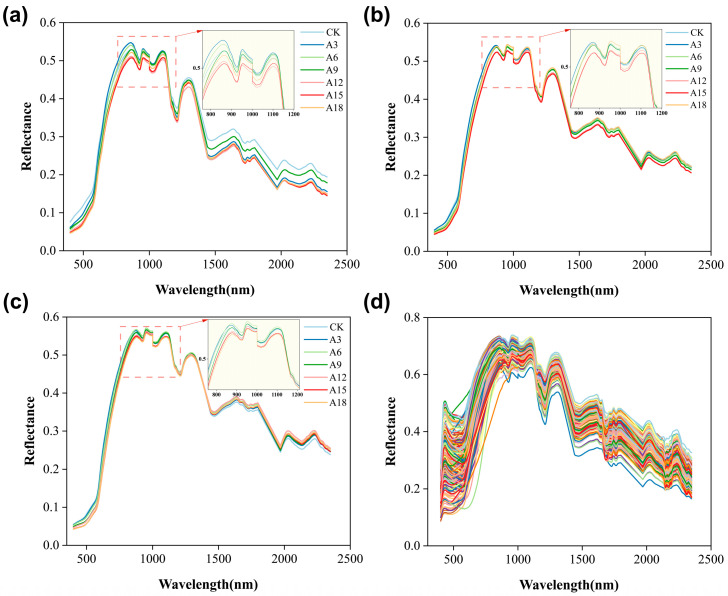
Average preprocessed spectra of all peanut samples. Note: (**a**–**c**) Spectral information of the three peanut varieties treated with aging, with line colors representing different aging durations. The spectral partial enlargement view focuses on the 760–1200 nm wavelength region. (**d**) Spectral information for untreated peanut varieties, with line colors denoting different peanut varieties.

Although the spectral data for each species showed a similar trend within the same spectral range, differences were observed in the reflectance amplitude, possibly owing to variations in the internal chemical components among the different seed varieties (Figure 1). This also indicated that the spectral data of peanut seeds exhibited variability, which was beneficial for the construction of monitoring models.

Although the noise-containing bands were manually removed, the high-dimensional spectral data still contained redundant and invalid information that could not be manually evaluated. Therefore, we used principal component analysis (PCA) to further process the data. PCA was used to reduce the high-dimensional data to 60 dimensions after comparing the model accuracy across different dimensions. The cumulative contributions of PCA1, PCA2, PCA3, PCA4, and PCA5, were 81.17%, 91.60%, 96.46%, 98.97%, and 99.59%, respectively (Figure S1).

**Figure 2:**
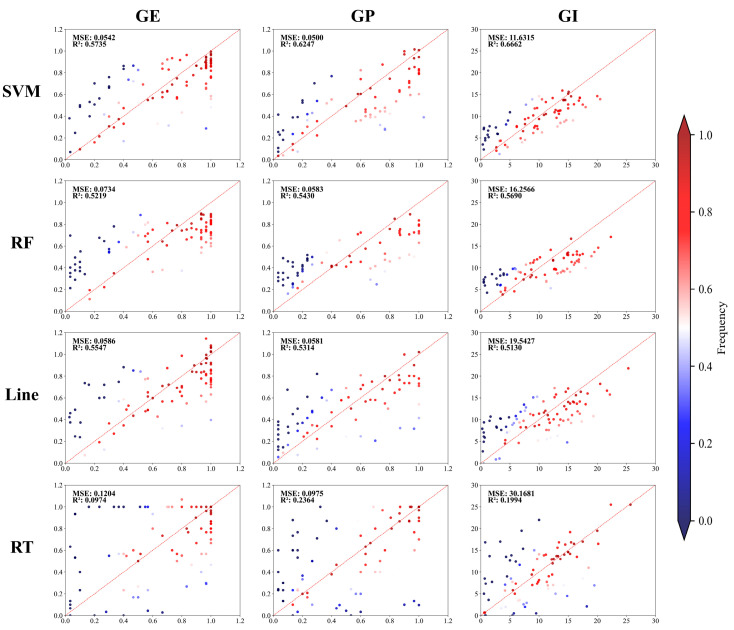
Correlation and scatterplot of true and predicted values. Note: The scatterplot color represents the distance from the points and the 1:1 diagonal. Red indicates a closer distance, while blue indicates a farther distance.

Vigor prediction models were constructed using 485 sets of vigor indexes and corresponding spectral data as input variables to assess the vigor levels of multiple seed varieties. Given the significant differences in the measured data during the analysis, four classical ML algorithms (SVM, RF, Line, and RT) were employed to build the models (Figure 2 and Table S1). For GE, the R^2^ values were 0.57 for SVM, 0.52 for RF, 0.55 for Line, and 0.097 for RT. For GP, the R^2^ values were 0.62 for SVM, 0.54 for RF, 0.53 for Line, and 0.24 for RT. For GI, the R^2^ values were 0.67 for SVM, 0.57 for RF, 0.51 for Line, and 0.19 for RT.

By observing the performance of various models, the RT algorithm struggled with the complexity of hyperspectral data. However, models employing the SVM, RF, and Line algorithms processed hyperspectral information more effectively, indicating their ability to accurately predict seed vigor. Subsequent analyses showed that the SVM and RF models were better suited for predicting candidate genes than the Line and RT models. The R^2^ values of the prediction models using the RT algorithm were lower than those of the other models, resulting in the inability to provide robust phenotypic prediction data. These findings highlight the potential of integrating hyperspectral data with ML models to effectively predict seed vigor.

### 2.3. Genes Regulating Seed Vigor Are Efficiently Mined Using GWAS

#### 2.3.1. Phenotypic Data Reflects Genetic Diversity

**Figure 3:**
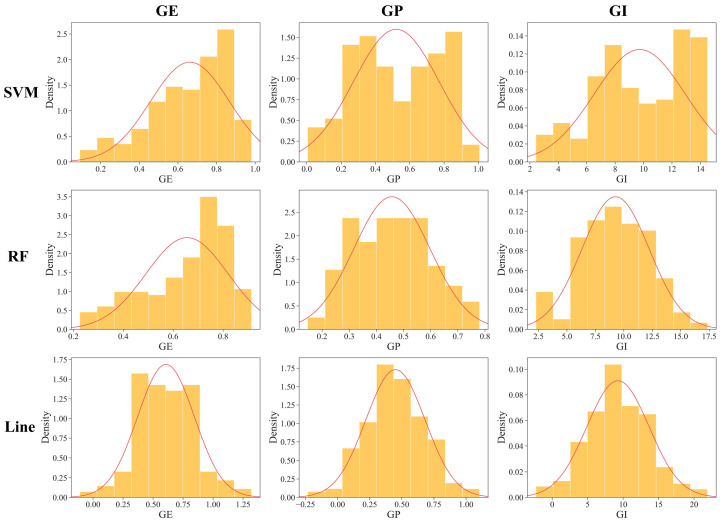
Distribution of predictive vigor indexes for model materials. The red curves represent the probability density function curves for each dataset. The vertical axis represents the probability density, while the horizontal axis denotes the values of the various indicators.

The predicted phenotypic values from ZC seeds are summarized and subsequently organized in Table 3. The predicted values of each indicator from the SVM, RF, and Line models were similar on average, suggesting consistent and overall vigor performance across the models. However, the coefficient of variation analysis revealed variability in the distribution of the predictive data for each model. The SVM and RF models showed more variability than Line. Additionally, the distribution of GE, GP, and GI was approximately normal (Figure 3). The SD analysis across the indexes revealed differences in vigor performance among the varying varieties, potentially providing more genetically diverse phenotypic materials for subsequent GWAS to better investigate differences at the genetic level.

#### 2.3.2. Screening of *Arahy.VMLN7L* and *Arahy.7XWF6F*

**Figure 4:**
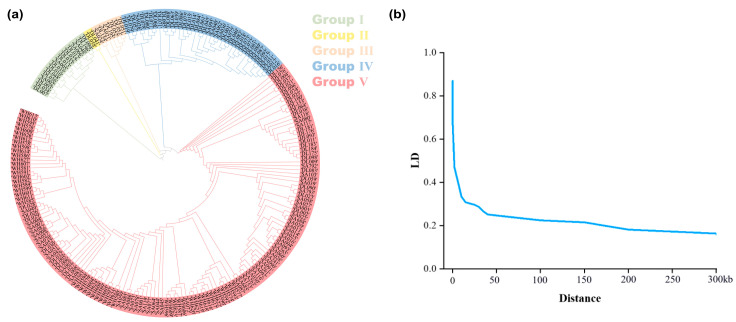
Population structure and linkage disequilibrium analysis. (**a**) Population structure grouping. (**b**) LD attenuation trend in the tested peanut varieties.

Owing to phenotypic differences primarily derived from diverse gene regulation, a detailed investigation of the functional genes associated with various predicted phenotypes was conducted. After pre-screening and filtering, 2,110,659 single nucleotide polymorphisms (SNPs) from 241 peanut varieties were obtained for subsequent association analysis. To investigate the origin and composition of each variety and to reduce false positives in subsequent GWAS analyses, Structure (v2.3.4) was employed to determine the optimal population structure. The relationship between K and ΔK indicated that the K-value was most likely five, suggesting the classification of the association group into five subgroups (Figure 4a).

**Figure 5:**
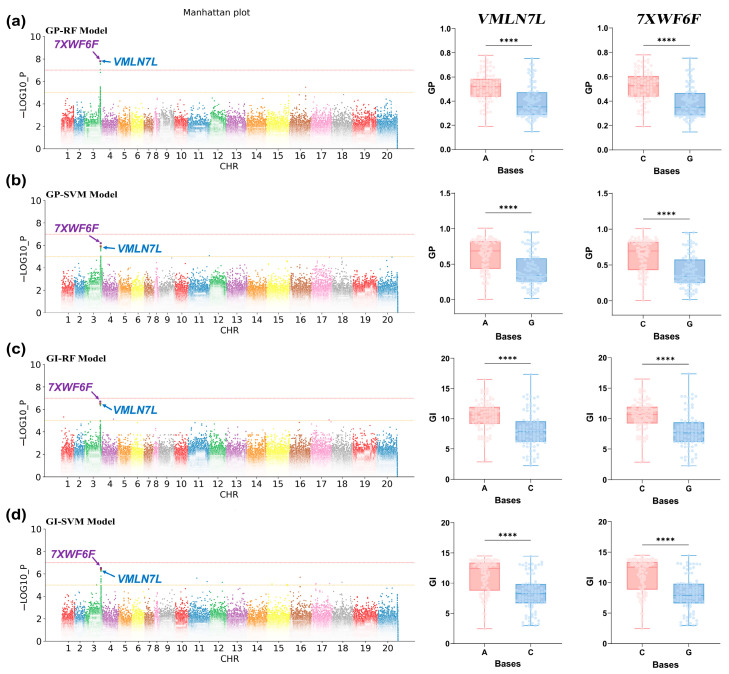
GWAS and haplotype analysis results for phenotypic predictive values. Note: (**a**–**d**) GWAS Manhattan plots (left) and haplotype analysis graphs (*VMLN7L* and *7XWF6F*) for phenotypic data predicted by GP-RF, GP-SVM, GI-RF, and GI-SVM, respectively. The horizontal axis is the chromosome number, and the vertical axis is −LOG10_P, which represents the *p* value calculated for each SNP as −log10. Different colors in the Manhattan plots represent SNPs from different chromosomes. Different colors in the haplotype analysis represent different haplotypes. Genes identified by different significant loci are indicated using color arrows. ****: significant correlation with *p* < 0.0001.

Based on phenotypic data from the SVM, RF, and Line models, GWAS was conducted using a mixed linear model. Subsequently, significant SNPs were screened with a threshold of *p* value < 1 × 10^−5^. A large number of significant SNPs were identified based on the GP and GI predictions from the SVM and RF models. However, significant loci were not better located when GE was used as phenotypic data for GWAS (Figure S4). Additionally, the predicted phenotypic data from the Line model did not facilitate a comprehensive exploration of the genetic relationship of seed vigor (Figure S4). To further identify SNPs associated with the vigor phenotype, we conducted a precise analysis and replicated localization using gene-based association (GBA) (Figure S2), ultimately identifying two significant SNPs in the GP and GI traits (Figure 5). SNP1 (Position: 125540699) and SNP2 (Position: 125601587) were discovered within the same QTL interval spanning approximately 100 kb. Moreover, these significant SNPs were identified through co-localization by both the SVM and RF models. In the 50-kb upstream and downstream regions of the two SNPs (Figure 4b), we identified numerous genes (Table S2). Haplotype analysis of these genes revealed that *Arahy.VMLN7L* and *Arahy.7XWF6F* were significantly associated with seed vigor levels (Figure 5, right). This suggests that both genes may be involved in the regulation of seed vigor in peanuts.

### 2.4. VMLN7L and 7XWF6F Are Involved in the Mechanisms Regulating Seed Vigor

**Figure 6:**
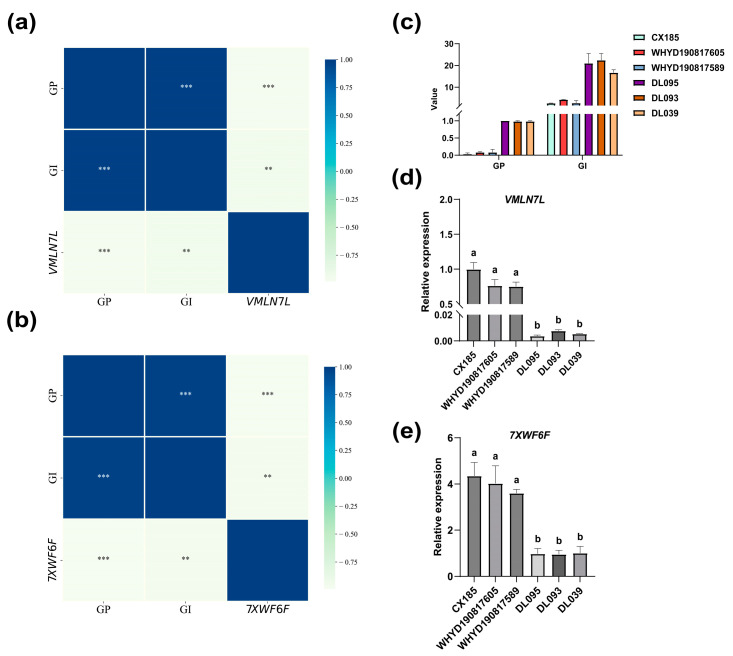
Relative expression of the *Arahy.VMLN7L* and *Arahy.7XWF6F* in seeds of different varieties and their correlation analysis with phenotypic traits. (**a**,**b**) Correlation analysis of *Arahy.VMLN7L* and *Arahy.7XWF6F* with the phenotype. (**c**) Actual vigor performance of high- and low-vigor germplasms. (**d**) Relative expression of *Arahy.VMLN7L* in varieties with different vigor levels. (**e**) Relative expression of *Arahy.7XWF6F* in varieties with different vigor levels. Different letters indicate significant differences based on one-way ANOVA of multiple tests (*p* < 0.05). **, ***: significant correlation with *p* < 0.01, *p* < 0.001.

To identify the function of the candidate genes, we explored their expression in different phenotypic groups and the potential physiological mechanisms in which they might be involved. Cluster analysis could effectively classify germplasms into high- and low-vigor categories, avoiding manual intervention (Figure S3). The haplotype differences between the high- and low-vigor peanut populations suggested that *Arahy.VMLN7L* and *Arahy.7XWF6F* influenced seed vigor (Figure 5). Gene expression could affect the performance of phenotypic traits. To better explore the gene expression behind the high- and low-vigor phenotypes, we selected three germplasms with very high vigor and three with very low vigor for qPCR analysis to examine *Arahy.VMLN7L* and *Arahy.7XWF6F* expression. Additionally, using three biological replicates ensured statistical significance and reduced the chance of random error. High-vigor varieties included DL095, DL093, and DL039, while low-vigor varieties included CX185, WHYD190817605, and WHYD190817589. Significant differences were observed in the actual GP and GI performance of the high- and low-vigor germplasms (Figure 6c). *Arahy.VMLN7L* and *Arahy.7XWF6F* were significantly expressed in the low-vigor germplasm but exhibited low expression in the high-vigor germplasm (Figure 6d,e). Moreover, the expression of these two genes was significantly correlated with GP and GI (Figure 6a,b).

The functions of these two genes were further analyzed using Blastp (Table S3). VMLN7L showed 47% amino acid sequence identity with LecRK-IX.1 (*AT5G10530*) in Arabidopsis thaliana. Both genes shared the same key domains, including the protein kinase domain (IPR000719), protein kinase-like domain (IPR011009), and legume lectin domain (IPR001220) [37], suggesting that they had potential functional similarities. In Arabidopsis, the kinase activity of LecRK-IX.1 could induce cell death [38]. Combining the qPCR results, we presumed that *Arahy.VMLN7L* may negatively regulate seed viability through the protein kinase domain, leading to cell death and consequently reducing seed vigor.

The expression product of *Arahy.7XWF6F* is Oligopeptide Transporter 4 (OPT4). 7XWF6F showed 78.53% amino acid sequence identity with AtOPT4 (*AT5G64410*) in Arabidopsis thaliana and shared the same conserved domain, OPT_sfam (IPR004813). Owing to the high homology between these two proteins, they largely perform the same functions. AtOPT4 has been identified as a glutathione transporter protein [39] that participates in glutathione metabolism. In the present study, the expression of *Arahy.7XWF6F* was observed at the early stage of germination in germplasm with high vigor, which is consistent with previous studies [40]. Moreover, previous research has found that AtOPT4 is prominently expressed in germinating Arabidopsis seeds and may play a role in nitrogen mobilization during seed germination and seedling development, likely providing nutrients and energy for growth and development. Therefore, we presume that 7XWF6F functions similarly to promote peanut seed germination and seedling growth. However, the relative expression of *Arahy.7XWF6F* was higher in the low-vigor germplasm than in the high-vigor germplasm in this study. This may be attributed to the seeds entering the after-ripening phase (AR phase) during dry storage and subsequently becoming dormant. To respond quickly to germination in the future, these AR-germplasms were capable of reserving material mobilization and energy production, including processes involving glutathione metabolism and amino acid metabolism [41]. However, low-vigor varieties are unable to germinate properly owing to internal influences during prolonged deep dormancy; however, *Arahy.7XWF6F* is still involved in glutathione metabolic activity. Conversely, high-vigor germplasms can rapidly break dormancy and enter a germination state. The related genes will display downregulated expression patterns after germination, and the seed coat is broken [41]. Therefore, the relative expression of *Arahy.7XWF6F* was higher in low-vigor varieties than in high-vigor varieties.

## 3. Discussion

### 3.1. Predictive Models Based on Vigor Phenotypes

Comparing this approach with that of a reported study [42], our study explored seed vigor across different varieties and aging periods utilizing a large natural population of peanuts. Vigor phenotypic and spectral data from 244 peanut seed types in their untreated state and several varieties under different aging treatments were analyzed using four ML algorithms to construct regression prediction models. Among these models, SVM and RF demonstrated better robustness in predicting seed vigor than Line and RT. Utilizing these predictive models enables the rapid acquisition of variation and characterization of seed vigor in different peanut varieties, thereby providing breeders with quick identification of ideal germplasm across a wide range of population materials. This finding holds significance for future research on high-vigor peanut varieties.

### 3.2. GWAS Results Confirm High-Throughput Predictive Phenotyping Validity in Investigating Genetic Relationships for Seed Vigor

To further assess the value of hyperspectral phenotypes in future molecular breeding, we performed a GWAS on GE, GP, and GI predicted by SVM, RF, and Line models to test whether these traits were effective in detecting genetic loci that regulate seed vigor. However, the Line model was not applicable for predicting genetic loci, probably owing to the accuracy or model construction. In the SVM and RF models, two significant SNPs were detected through GP and GI. Compared to GE, GP, and GI may be more suitable for mining loci related to vigor. After screening by GBA with haplotype analysis, we identified *Arahy.VMLN7L* and *Arahy.7XWF6F*. To further investigate these two genes, we performed qPCR to detect the expression of both genes in high- and low-vigor varieties. Further BLAST and protein structural domain analyses revealed that VMLN7L shares the key kinase structural domain with LecRK-IX.1 in Arabidopsis. This kinase structural domain could induce cell death. Moreover, *Arahy.VMLN7L* exhibited higher expression in low-vigor germplasms. Therefore, it is highly likely that *Arahy.VMLN7L* impairs the germination mechanism by inducing the death of the endosperm, embryo cells, or other critical cells in peanut seeds, rendering the germplasm less viable and causing the seeds to fail to germinate under suitable conditions. This suggests that *Arahy.VMLN7L* can negatively regulate seed vigor. The *Arahy.7XWF6F* expression product was OPT4, which was highly homologous to and shared the same conserved structural domain as AtOPT4 (*AT5G64410*) in Arabidopsis, indicating similar functions. *Arahy.7XWF6F* expression was observed at the early stage of germination in the highly viable germplasm. Hence, 7XWF6F is likely to be involved in nitrogen mobilization during seed germination and seedling development, similar to the function of AtOPT4, accelerating organic matter metabolism and providing an energy base for seed germination. The cotyledons hold most of the stored nutrients, and the degradation of bulk stored proteins occurs during the post-germination period [43]. Therefore, as an oligopeptide transporter protein, 7XWF6F may also be involved in the transport of peptides derived from the degradation of storage proteins in growing and dividing cells, as well as in the mobilization of peptides from the cotyledons to the embryonic axes during the post-germination period to promote the development of young shoots. These results strongly suggest that phenotypic information obtained from hyperspectral monitoring can be used for the genetic dissection of seed vigor performance. Additionally, this demonstrates that genetic loci co-localized based on different traits and models may be more valuable for research. 

High-vigor seeds exhibit superior field performance, including high vigor, high seedling emergence, and uniform seedling growth. Additionally, high-vigor seeds possess significant growth advantages and production potential, enabling them to overcome unfavorable environmental conditions and thereby directly or indirectly enhance field yield [44]. Our study validated the feasibility of using remote sensing phenotypes to identify functional genes in peanut germplasm and inferred the effective pathways of *Arahy.VMLN7L* and *Arahy.7XWF6F* in regulating seed vigor through homology, protein structural domain, and expression analyses. The methods and results presented in this study provide valuable gene loci for future breeding efforts to develop high-vigor peanut germplasms, significantly reducing the time and resources required for traditional phenotypic selection, thereby alleviating researchers’ workload and accelerating the breeding process. Furthermore, seed vigor is a complex and comprehensive agronomic trait, and our study demonstrated that hyperspectral remote sensing technology can effectively predict seed vigor levels, thereby supporting the breeding process. Moreover, the methodology employed in this study is generalizable and may be extended to other crops in the future, expanding its impact on molecular breeding efforts and agricultural productivity.

### 3.3. Constraints and Potential Future Research

Each research method has some limitations that require further optimization in the future. Although hyperspectral spectroscopy possesses some penetration ability, the outer seed coat of the peanut kernel still interferes with the monitoring process. Overcoming the effect caused by the seed coat is essential for improving the accuracy of the model in the future. It is well known that seed vigor is a complex and comprehensive genetic trait, which undoubtedly impacts the effectiveness of model monitoring and GWAS analysis. Additionally, enhancing model fitting can reduce the gap between predicted and measured phenotypes, affecting GWAS results. Nevertheless, accurately identifying functional genes also depends on factors such as the proportion of characteristic phenotype data, population size, population structure, and LD analysis [45]. In this study, extensive static phenotypic data from various peanut varieties were used to construct models and mine functional genes using GWAS. This approach may have overlooked certain genes. Therefore, further research analyses may require additional phenotypic and spectral data from seeds at various growth phases, including initial seedling morphology and diverse genotype populations. Additionally, large-scale implementation of hyperspectral remote sensing requires significant investment in equipment and expertise, posing a challenge for smaller breeding programs or resource-limited environments.

Combining the above limitations with the existing research results, future research should focus on the following aspects. In terms of remote sensing technology, optical instruments require further reform to enhance the penetration ability of the hyperspectral spectrum and attenuate surface substances’ interference on detection, thereby improving the accuracy of monitoring the internal plants’ substances. Regarding functional genes, identifying more gene loci in the exploration of functional genes can significantly expand future breeding efforts. Achieving this goal may require access to extensive phenotypic data from seeds at various growth stages, physiological states, and across varieties, including initial seedling morphology and diverse genotypic populations. Moreover, developing algorithms capable of exploring the optimal combination of population structure and genotyping may improve GWAS analyses and expand the number of gene loci. In future studies, advanced genomic technologies should be integrated to construct a comprehensive genetic map to facilitate the identification of more functional gene loci. Future studies should emphasize the development of cost-effective remote sensing solutions applicable to a broader range of breeding programs, including the utilization of low-cost drones, simplified imaging systems, and user-friendly data analysis software. While these solutions require further development, our study has confirmed the effectiveness of mining functional genes based on remotely sensed phenotypes at this stage, establishing a crucial foundation for future advancements. In the future, we hope to expand our study to a broader range of species to provide more comprehensive insights and applications.

## 4. Materials and Methods

### 4.1. Seed Materials and Experimental Design

The seed experiments were designed to compare seed vigor across different varieties and performance under different physiological states. Natural populations of peanuts, comprising 247 varieties, were collected from various global regions and subsequently harvested from the Hainan and Guangdong provinces in China. The seeds were divided into two groups: untreated (244 varieties, 244 samples) and aging (3 varieties, 84 samples). There were 30 seeds per sample. The untreated seed samples were sun-dried after harvesting and stored in a low-temperature environment. Shelling of peanut seeds was conducted before all experiments were initiated, ensuring that the interval between shelling and subsequent experiments did not exceed 24 h. Aging treatments for each variety were divided into seven aging periods: 0d (CK), 3d (A3), 6d (A6), 9d (A9), 12d (A12), 15d (A15), and 18d (A18), with four replicates per aging period. Setting a gradient of aging time was intended to better observe the dynamic vigor changes of peanut seeds at varying degrees of aging.

### 4.2. Spectral Measurements

In this study, the hyperspectral information of all seed samples was acquired with an ASD Field-Spec 4 Hi-Res (ASD; Malvern Panalytical Ltd., Malvern, Worcestershire, UK) using a halogen lamp for illumination in a dark environment. The ASD Field-Spec 4 Hi-Res is capable of delivering stable and reliable spectral performance over the full range of the solar irradiation spectrum (350–2500 nm). The ASD was preheated for approximately 30 min to stabilize its internal system. Subsequently, the halogen light source and probe were adjusted to approximately 37 cm and 17 cm from the tabletop. After using RS^3^ (v6.0, Malvern Panalytical Ltd., Malvern, Worcestershire, UK) to control the ASD for calibrating the whiteboard, the seed samples were uniformly arranged in the vessel for measurement. The spectral information of the 30 seeds in each sample was concurrently collected as the spectral data of the sample. Different seed treatments utilized different measurement frequencies and methods. Untreated seeds were measured in two sets of 170 and 235 samples, containing 161 replicates, with five measurements per sample. The aged seeds were measured in 84 samples, each from four different angles (five times per angle), to enhance the accuracy of the spectral information regarding vigor changes. The measurements for each sample were averaged to obtain the spectral data for that sample.

**Figure 7:**
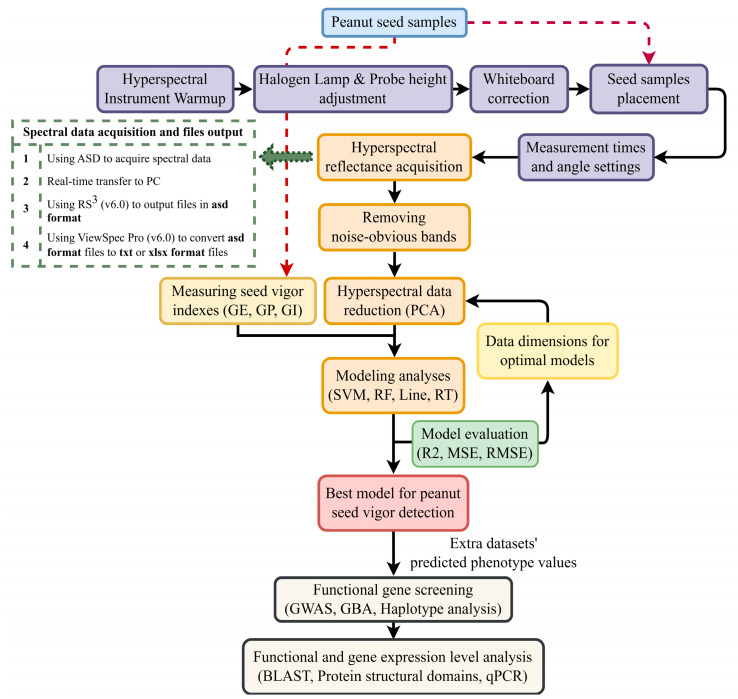
Research process of this study. The black arrows represent the normal flow of the experiment. The red arrows represent using seed samples as test material. The green arrow represents more detailed procedure information.

The original format of all peanut spectral data measured by the ASD was the asd format. When the ASD collected the spectral reflectance of a seed sample, the spectral information was transferred in real time to RS^3^ (v6.0) on a PC, and a file in asd format was used as the output. The files contained the spectral information data of the seed samples. Since the data distribution of this format file was not easy to process directly, we used ViewSpec Pro (v6.0, Malvern Panalytical Ltd., Malvern, Worcestershire, UK) to convert the asd file to a txt or an xlsx format file to facilitate subsequent data observation, processing, and analysis (Figure 7).

### 4.3. Seed Vigor Data Acquisition

Spectral data were collected and a germination test was conducted at the College of Agriculture, South China Agricultural University (Guangzhou, China). To increase the sample dataset for models and minimize experimental error, untreated peanuts underwent two germination tests (N1 and N2) for 4d, while aging seeds were tested for 6d.

All peanut samples were initially placed in dry germination boxes covered with germination paper. After the measured quantity of ultrapure water was added, the boxes were sealed and positioned in a light-free environment within a culture chamber. Germination progress was evaluated by observing the emergence of white sprouts on the seeds. Additionally, daily counts of germinated seeds were conducted, and the water in the germination boxes was replaced regularly.

At the end of the test, vigor indexes were calculated using the following formula:

(1) $$\mathrm{Germination}\;\mathrm{rate}\;\left(\mathrm{GE}\right)=\frac{n_{1}}{N}\times 100\%$$

(2) $$\mathrm{Germination}\;\mathrm{potential}\;\left(\mathrm{GP}\right)=\frac{n_{2}}{N}\times 100\%$$

(3) $$\mathrm{Germination}\;\mathrm{index}\;\left(\mathrm{GI}\right)=\sum \frac{G_{t}}{D_{t}}$$

where n_1_ denotes the total number of seeds germinated at the end of the germination test, n_2_ denotes the number of seeds germinated when the daily germination rate peaked (2d in this study), N is the total number of seed samples, Gt represents the daily count of germinated seeds, and Dt is the number of days to germination.

### 4.4. Hyperspectral Data Pre-Processing

The measured spectral data contained a significant amount of noise. Therefore, the spectral regions spanning 350–400 nm, 1340–1440 nm, 1800–1970 nm, and 2349–2500 nm were manually removed during data processing. Consequently, the preprocessed spectral data comprised 1680 features. Moreover, the remaining data were averaged, resulting in 485 hyperspectral sets. Although high-dimensional data contained valuable feature information related to seed vigor, they also included various sources of noise, redundant information, and irrelevant features that cannot be manually observed and processed. These factors contributed to the poor accuracy of the model and hindered the precise prediction of peanut seed vigor indexes.

PCA is a popular method for reducing dimensionality and simplifying high-dimensional data to fewer-dimensional data while retaining the valid information of the original data [46]. This approach can effectively address the challenge of manually identifying worthless information, enhancing the algorithm’s operation and the model’s predictive ability. Selecting the appropriate principal components is crucial for retaining valid information [47]. In this study, the PCA function was used from Sklearn to reduce the spectral data dimensions to 50, 60, 100, and 120, constructing models for each vigor index. Comparing the models (Figure 7), those based on 60 dimensions exhibited the most effective prediction of peanut seed vigor.

### 4.5. Modeling Approaches

**Figure 8:**
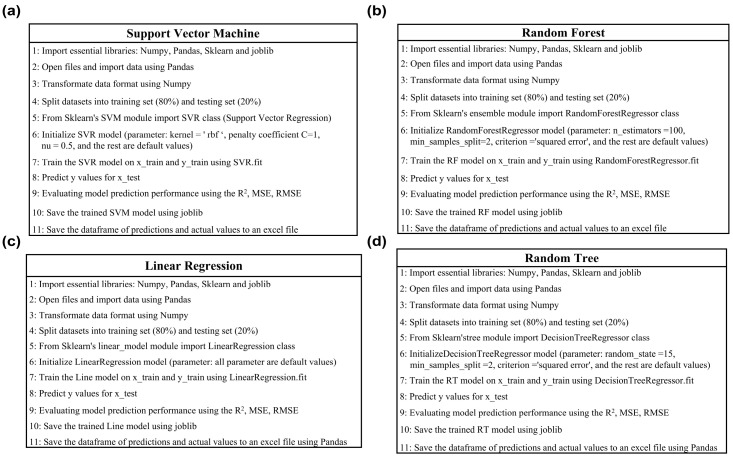
Machine learning algorithms for seed vigor prediction models. (**a**) SVM model. (**b**) RF model. (**c**) Line model. (**d**) RT model.

Four regression models were constructed for more efficient prediction of peanut seed vigor: SVM, RF, Line, and RT, using the Sklearn, Pandas, OS, Numpy, Seaborn, and Matplotlib libraries in Python 3.8 (Figure 8). SVM maps spectral data to a feature space and uses linear solving to determine a hyperplane, effectively addressing regression challenges [48]. RT comprises a single CART, while RF randomly selects training samples to construct multiple CARTs, and their average output serves as the final prediction value [49]. Line is a simple regression model type seeking a linear functional relationship between variables to predict unknown variables. The dependent variables GE, GP, and GI were used in this study to build the four regression models (SVM, RF, Line, and RT).

The following details the parameter settings and training process for each model. The 485 sets of phenotypic and spectral data were employed as the datasets. First, the dataset was randomly divided into training and validation sets in an 8:2 ratio. To train the SVM regression model, we selected the radial basis function (RBF) as the kernel function. The SVM model was initialized with parameters such as the penalty coefficient C (default value 1) to control the model’s tolerance to training errors, the kernel function coefficient gamma (default value being the inverse of the feature dimensions) to determine the range of influence of the RBF kernel function, and parameter nu (default value 0.5) to set the upper limit of the training error and the lower limit of the support vector. Finally, the fitting method of the SVR function was applied to train the model with the labeled and feature values of the training data, enabling it to learn the data patterns and find the optimal support vectors and decision boundaries, thereby achieving regression predictions on new data.

In the RF model, performance optimization was achieved by setting several parameters. The model used n_estimators (default value of 100) to control for the number of trees. The parameter max_depth controlled the maximum depth of each tree, the min_samples_split determined the minimum number of samples for node splitting, and the min_samples_leaf controlled the minimum number of samples for each leaf node. All parameters were set to their default values without making special changes. Additionally, the model used the square error as a partition criterion to reduce the variance of the feature selection criterion and minimized the L2 loss using the mean value of each terminal node. Finally, the fit method in the Random Forest Regressor function was applied to train the model. This method improved the accuracy and stability of the model by constructing multiple decision trees, using bootstrap sampling and random selection of features, and combining the predictions of all the trees through averaging.

In the Line model, we used default values for all parameters. The model was trained using the fit method of the Linear Regression function. The best regression coefficients were found using the least squares method, allowing the model to accurately describe the linear relationship between the input features and the target values in the training data. These regression coefficients were stored in the model object for future prediction.

In the RT model, we initialized a decision tree regression model and set the random state to 15 to ensure result repeatability. The random state controlled the random number generator, ensuring consistent results across multiple runs. All other parameters were set to their default values. Finally, the fit method of the Decision Tree Regressor function was applied to train the model. This method built a decision tree by recursively splitting the data and selecting the best split points to learn the patterns in the training data, ultimately achieving prediction.

The best model was selected based on evaluation metrics, including r-squared (R^2^), mean squared error (MSE), and root mean square error (RMSE) from SVM, RF, Line, and RT. Regression models with higher R^2^ values and lower MSE and RMSE demonstrated superior performance and accuracy.

The relevant formulas are as follows:

(4) $$R^{2}=1-\frac{\sum_{i=1}^{n}{\left(\hat{y_{t}}-y_{i}\right)}^{2}}{\sum_{i=1}^{n}{\left(\overset{-}{y}-y_{i}\right)}^{2}}$$

(5) $$\mathrm{MSE}=\frac{1}{n}\sum_{i=1}^{n}{\left(\hat{y_{i}}-y_{i}\right)}^{2}$$

(6) $$\mathrm{RMSE}=\sqrt{\frac{1}{n}\sum_{i=1}^{n}{\left(\hat{y_{i}}-y_{i}\right)}^{2}}$$

where n denotes the number of samples, y_i_ represents the actual value of the vigor index, and ŷ_i_ is the predicted value.

### 4.6. Vigor Phenotype and Genetics Analysis

#### 4.6.1. Phenotypic Statistical Analysis

Upon comparing the four models, the RT models exhibited a subpar fit among the variables, rendering them ineffective in predicting peanut seed vigor. The phenotypic data included predicted vigor index values from the SVM, RF, and Line models. To validate the models and GWAS results, a separate set of spectral data called ZC (191 natural peanut populations harvested from Zengcheng District, Guangzhou, China), which was not used in the modeling process, served as the input dataset for the models. This led to a phenotypic dataset for each model comprising predicted three vigor indicators’ phenotypic information (573 datasets in total). Moreover, descriptive analysis was conducted on the predictive vigor indicator values from the three models.

#### 4.6.2. Genotyping

To explore the genetic relationships between peanuts with varying vigor levels across different varieties, 241 peanut varieties were genotyped, and quality control of the genotype data was conducted using PLINK (v1.9) [50]. Markers with missing rates > 0.1 or minor allele frequencies < 0.05 were excluded.

#### 4.6.3. Population Structure and Linkage Disequilibrium Analysis

Structure (v2.3.4) [51] was used to determine the optimal K-value for population structure analysis. Additionally, a phylogeny figure was plotted using iTOL (https://itol.embl.de/, accessed on 14 July 2024). Furthermore, linkage disequilibrium (LD) analysis was conducted using TASSEL (v5.0) [52]. LD attenuation distance between SNP pairs was estimated using r^2^ (squared correlation between two loci) and D’ (standardized disequilibrium coefficient).

#### 4.6.4. GWAS Analysis

GWAS analysis was conducted utilizing the mixed linear model in EMMAX [53]. SNPs with a *p* value < 1 × 10^−5^ (−log10 (*p*) > 5) were retained, and candidate genes within 50 kb upstream and downstream regions of significant SNPs were identified. The reference genome used for genetic annotation was *arahy.Tifrunner.gnm1.KYV3.genome_main.fna* [54]. Manhattan and quantile-quantile plots were created using Python 3.8 and other toolkits (NumPy, Pandas, Seaborn, and Matplotlib) to visualize the results. All operations were performed using a Linux system.

#### 4.6.5. GBA and Haplotype Analysis

Gene-based association (GBA) focuses on the overall level of a gene, which can improve localization accuracy, reduce false positives, and identify rare genetic variants [55,56,57], thereby enhancing the ability to understand the genetic basis of complex traits. To accurately screen genes, we performed GBA analysis of GWAS-screened SNPs using EMMAX (v0~beta.20100307-5) and RStudio (v2024.04.1-748). Furthermore, the screened genes were subjected to haplotype analysis using Python 3.8, to assess their potential impact on the vigor phenotype of the seed population. Data were analyzed using GraphPad Prism 10 (v10.0; GraphPad Software, Boston, MA, USA).

#### 4.6.6. Expression Analysis of Candidate Genes

Clustering analysis was performed separately for N1 and N2 using the K-means algorithm. Varieties appearing in the same category in both analyses were selected as candidate materials. From these, extreme phenotypic germplasm with high and low vigor were subsequently identified, with each category containing three varieties.

The selected germplasm underwent a four-day immersion germination experiment. The germinated seed parts were used as the material for analysis. RNA was extracted for gene expression analyses using the FastPure Universal Plant Total RNA Isolation Kit (Vazyme Biotech Co., Ltd., Nanjing, China). Reverse transcription was performed using HiScript III RT Super-Mix for qPCR (+gDNA wiper). Subsequently, real-time fluorescence quantitative PCR was performed using ChamQ Universal SYBR qPCR premix and specific primers (Table 4). The relative gene expression was calculated based on 2^−ΔΔCt^ [58], with *Actin* as an internal reference gene. Data were analyzed using GraphPad Prism 10.

## 5. Conclusions

The findings revealed that RF and SVM models were the most effective ML algorithms for obtaining reliable vigor phenotypic information, capable of detecting genes potentially associated with vigor performance. Furthermore, GP and GI may be more suitable for exploring candidate genes that regulate seed vigor. GWAS, GBA, haplotype, and qPCR analyses revealed candidate genes regulating seed vigor, including *Arahy.VMLN7L* and *Arahy.7XWF6F*. In conclusion, this study not only provides directions for future breeding efforts to cultivate high-vigor germplasm but also verifies the validity of combining GWAS with hyperspectral phenotypic information to explore the genetic basis of seed vigor.

## Figures and Tables

**Table 1:** Statistics of measured phenotypic data from all germination experiments.

Vigor Index	Experiments	Max	Min	Mean	SD	CV
	N1	1.0000	0.0278	0.8217	0.2501	
	N2	1.0000	0.0000	0.5105	0.3563	
	CK	1.0000	0.3667	0.8194	0.2646	
	A3	1.0000	0.0667	0.7000	0.3618	
GE	A6	1.0000	0.0333	0.6194	0.3875	0.5516
	A9	0.8000	0.0000	0.3472	0.2684	
	A12	0.5000	0.0000	0.2000	0.1933	
	A15	0.3667	0.0000	0.0944	0.1294	
	A18	0.2667	0.0000	0.0611	0.0908	
	N1	1.0000	0.0000	0.7284	0.2967	
	N2	1.0000	0.0000	0.2875	0.2732	
	CK	0.9667	0.2333	0.7667	0.2689	
	A3	0.9667	0.0000	0.6194	0.3751	
GP	A6	0.9000	0.0333	0.4833	0.3103	0.5839
	A9	0.5000	0.0000	0.1833	0.1709	
	A12	0.2000	0.0000	0.0667	0.0752	
	A15	0.1667	0.0000	0.0194	0.0481	
	A18	0.1000	0.0000	0.0167	0.0333	
	N1	25.5000	0.5000	13.1971	4.9501	
	N2	19.3333	0.0000	6.4408	4.9333	
	CK	28.8333	6.8333	22.3708	8.5257	
	A3	26.7000	0.6667	16.4833	10.0378	
GI	A6	21.9167	0.5000	12.3347	8.1685	0.5683
	A9	11.2500	0.0000	4.4361	3.7211	
	A12	5.0333	0.0000	2.2736	2.2277	
	A15	4.1500	0.0000	0.9153	1.3953	
	A18	2.9167	0.0000	0.6056	0.9658	

**Table 2:** Detailed statistics of data from aging germination experiments.

		GE			GP			GI	
	P1 *	P2 *	P3 *	P1	P2	P3	P1	P2	P3
CK	0.4750	0.9833	0.9333	0.4250	0.9500	0.8667	27.7583	11.3750	23.0917
A3	0.2167	0.9500	0.9333	0.1250	0.8667	0.8667	22.8417	3.5167	23.0917
A6	0.1083	0.8333	0.9167	0.0833	0.6250	0.7417	17.4250	1.8417	17.7375
A9	0.0500	0.6250	0.3667	0.0167	0.3250	0.2083	7.7917	0.5708	4.9458
A12	0.0000	0.3833	0.2167	0.0000	0.1167	0.0833	4.2833	0.0000	2.5375
A15	0.0000	0.2250	0.0583	0.0000	0.0500	0.0083	2.3250	0.0000	0.4208
A18	0.0000	0.1583	0.0250	0.0000	0.0500	0.0000	1.6417	0.0000	0.1750

* P1, P2 and P3 refer to three peanut varieties.

**Table 3:** Descriptive statistics of predictive seed vigor.

Vigor Index	Models	Max	Min	Mean	SD	CV	Skewness	Kurtosis
GE	SVM	0.9809	0.0920	0.6621	0.2046	0.3090	−0.7971	−0.1186
	RF	0.9138	0.2260	0.6556	0.1647	0.2512	−0.7352	−0.4438
	Line	1.3201	−0.1096	0.6078	0.2362	0.3886	0.1031	0.3570
GP	SVM	1.0066	0.0055	0.5213	0.2498	0.4792	−0.0525	−1.2257
	RF	0.7797	0.1476	0.4570	0.1407	0.3078	0.1131	−0.8223
	Line	1.1046	−0.2320	0.4500	0.2304	0.5120	0.0076	0.0389
GI	SVM	14.5042	2.4231	9.7315	3.1941	0.3282	−0.3235	−0.9603
	RF	17.3580	2.2775	9.2745	2.9555	0.3187	−0.0926	−0.3725
	Line	21.9095	−2.2886	9.2753	4.3806	0.4723	0.1001	0.0957

**Table 4:** Primers used for qPCR.

Gene	Forward Primer (5′-3′)	Reverse Primer (5′-3′)
*Arahy.VMLN7L*	CCCATGATGCGCCACAAAAT	CGGTGAAATTCTTACCGCCA
*Arahy.7XWF6F*	ATGGGTTTTGGTTGGAACGG	CTATGCCCTTAGCAGTGGGA
*Actin*	GATTGGAATGGAAGCTGCTG	CGGTCAGCAATACCAGGGAA

## Data Availability

The original contributions presented in the study are included in the article and Supplementary Materials; further inquiries can be directed to the corresponding author.

## Supplementary Materials

The following supporting information can be downloaded at: https://www.mdpi.com/article/10.3390/ijms25158414/s1.

## References

[1] Yu H., Liu H., Erasmus S.W., Zhao S., Wang Q., Van Ruth S.M. (2020). Rapid High-Throughput Determination of Major Components and Amino Acids in a Single Peanut Kernel Based on Portable near-Infrared Spectroscopy Combined with Chemometrics. Ind. Crop. Prod..

[2] Hosseini Taheri S.E., Bazargan M., Rahnama Vosough P., Sadeghian A. (2024). A Comprehensive Insight into Peanut: Chemical Structure of Compositions, Oxidation Process, and Storage Conditions. J. Food Compos. Anal..

[3] Mahtta R., Fragkias M., Güneralp B., Mahendra A., Reba M., Wentz E.A., Seto K.C. (2022). Urban Land Expansion: The Role of Population and Economic Growth for 300+ Cities. NPJ Urban Sustain..

[4] Prăvălie R., Patriche C., Borrelli P., Panagos P., Roșca B., Dumitraşcu M., Nita I.-A., Săvulescu I., Birsan M.-V., Bandoc G. (2021). Arable Lands under the Pressure of Multiple Land Degradation Processes. A Global Perspective. Environ. Res..

[5] Wang X., Zheng H., Tang Q., Mo W., Ma J. (2019). Effects of Gibberellic Acid Application after Anthesis on Seed Vigor of Indica Hybrid Rice (*Oryza sativa* L.). Agronomy.

[6] Reed R.C., Bradford K.J., Khanday I. (2022). Seed Germination and Vigor: Ensuring Crop Sustainability in a Changing Climate. Heredity.

[7] Finch-Savage W.E., Bassel G.W. (2016). Seed Vigour and Crop Establishment: Extending Performance beyond Adaptation. J. Exp. Bot..

[8] Rajjou L., Duval M., Gallardo K., Catusse J., Bally J., Job C., Job D. (2012). Seed Germination and Vigor. Annu. Rev. Plant Biol..

[9] Li H., Yue H., Xie J., Bu J., Li L., Xin X., Zhao Y., Zhang H., Yang L., Wang J. (2021). Transcriptomic Profiling of the High-Vigour Maize (*Zea mays* L.) Hybrid Variety Response to Cold and Drought Stresses during Seed Germination. Sci. Rep..

[10] Zhang T., Fan S., Xiang Y., Zhang S., Wang J., Sun Q. (2020). Non-Destructive Analysis of Germination Percentage, Germination Energy and Simple Vigour Index on Wheat Seeds during Storage by Vis/NIR and SWIR Hyperspectral Imaging. Spectrochim. Acta A Mol. Biomol. Spectrosc..

[11] Al-Turki T.A., Baskin C.C. (2017). Determination of Seed Viability of Eight Wild Saudi Arabian Species by Germination and X-ray Tests. Saudi J. Biol. Sci..

[12] Hill H.J., Taylor A.G., Huang X.L. (1988). Seed Viability Determinations in Cabbage Utilizing Sinapine Leakage and Electrical Conductivity Measurements. J. Exp. Bot..

[13] Fenollosa E., Jené L., Munné-Bosch S. (2020). A Rapid and Sensitive Method to Assess Seed Longevity through Accelerated Aging in an Invasive Plant Species. Plant Methods.

[14] Gnyp M.L., Miao Y., Yuan F., Ustin S.L., Yu K., Yao Y., Huang S., Bareth G. (2014). Hyperspectral Canopy Sensing of Paddy Rice Aboveground Biomass at Different Growth Stages. Field Crop. Res..

[15] Stroppiana D., Boschetti M., Brivio P.A., Bocchi S. (2009). Plant Nitrogen Concentration in Paddy Rice from Field Canopy Hyperspectral Radiometry. Field Crop. Res..

[16] Mishra P., Lohumi S. (2021). Improved Prediction of Protein Content in Wheat Kernels with a Fusion of Scatter Correction Methods in NIR Data Modelling. Biosyst. Eng..

[17] Hu N., Li W., Du C., Zhang Z., Gao Y., Sun Z., Yang L., Yu K., Zhang Y., Wang Z. (2021). Predicting Micronutrients of Wheat Using Hyperspectral Imaging. Food Chem..

[18] Zhao D., Raja Reddy K., Kakani V.G., Read J.J., Carter G.A. (2003). Corn (*Zea mays* L.) Growth, Leaf Pigment Concentration, Photosynthesis and Leaf Hyperspectral Reflectance Properties as Affected by Nitrogen Supply. Plant Soil.

[19] Cao C., Wang T., Gao M., Li Y., Li D., Zhang H. (2021). Hyperspectral Inversion of Nitrogen Content in Maize Leaves Based on Different Dimensionality Reduction Algorithms. Comput. Electron. Agric..

[20] Gao C., Li H., Wang J., Zhang X., Huang K., Song X., Yang W., Feng M., Xiao L., Zhao Y. (2024). Combined Use of Spectral Resampling and Machine Learning Algorithms to Estimate Soybean Leaf Chlorophyll. Comput. Electron. Agric..

[21] Sun J., Shi X., Zhang H., Xia L., Guo Y., Sun X. (2019). Detection of Moisture Content in Peanut Kernels Using Hyperspectral Imaging Technology Coupled with Chemometrics. J. Food Process. Eng..

[22] Xing M., Long Y., Wang Q., Tian X., Fan S., Zhang C., Huang W. (2023). Physiological Alterations and Nondestructive Test Methods of Crop Seed Vigor: A Comprehensive Review. Agriculture.

[23] Kovar M., Brestic M., Sytar O., Barek V., Hauptvogel P., Zivcak M. (2019). Evaluation of Hyperspectral Reflectance Parameters to Assess the Leaf Water Content in Soybean. Water.

[24] Yi Q.-X., Huang J.-F., Wang F.-M., Wang X.-Z., Liu Z.-Y. (2007). Monitoring Rice Nitrogen Status Using Hyperspectral Reflectance and Artificial Neural Network. Environ. Sci. Technol..

[25] Zhang L., An D., Wei Y., Liu J., Wu J. (2022). Prediction of Oil Content in Single Maize Kernel Based on Hyperspectral Imaging and Attention Convolution Neural Network. Food Chem..

[26] Zhang D., Zhang J., Peng B., Wu T., Jiao Z., Lu Y., Li G., Fan X., Shen S., Gu A. (2023). Hyperspectral Model Based on Genetic Algorithm and SA-1DCNN for Predicting Chinese Cabbage Chlorophyll Content. Sci. Hortic..

[27] Strachan I.B., Pattey E., Boisvert J.B. (2002). Impact of Nitrogen and Environmental Conditions on Corn as Detected by Hyperspectral Reflectance. Remote Sens. Environ..

[28] Lu B., Dao P.D., Liu J., He Y., Shang J. (2020). Recent Advances of Hyperspectral Imaging Technology and Applications in Agriculture. Remote Sens..

[29] Uffelmann E., Huang Q.Q., Munung N.S., de Vries J., Okada Y., Martin A.R., Martin H.C., Lappalainen T., Posthuma D. (2021). Genome-Wide Association Studies. Nat. Rev. Methods Primers.

[30] Korte A., Farlow A. (2013). The Advantages and Limitations of Trait Analysis with GWAS: A Review. Plant Methods.

[31] Tam V., Patel N., Turcotte M., Bossé Y., Paré G., Meyre D. (2019). Benefits and Limitations of Genome-Wide Association Studies. Nat. Rev. Genet..

[32] Barik S.R., Pandit E., Sanghamitra P., Mohanty S.P., Behera A., Mishra J., Nayak D.K., Bastia R., Moharana A., Sahoo A. (2022). Unraveling the Genomic Regions Controlling the Seed Vigour Index, Root Growth Parameters and Germination per Cent in Rice. PLoS ONE.

[33] Morris K., Barker G.C., Walley P.G., Lynn J.R., Finch-Savage W.E. (2016). Trait to Gene Analysis Reveals That Allelic Variation in Three Genes Determines Seed Vigour. New Phytol..

[34] Chen Z., Lancon-Verdier V., Le Signor C., She Y.-M., Kang Y., Verdier J. (2021). Genome-Wide Association Study Identified Candidate Genes for Seed Size and Seed Composition Improvement in *M*. truncatula. Sci. Rep..

[35] Dai L., Lu X., Shen L., Guo L., Zhang G., Gao Z., Zhu L., Hu J., Dong G., Ren D. (2022). Genome-Wide Association Study Reveals Novel QTLs and Candidate Genes for Seed Vigor in Rice. Front. Plant Sci..

[36] Liu Y., Shao L., Zhou J., Li R., Pandey M.K., Han Y., Cui F., Zhang J., Guo F., Chen J. (2022). Genomic Insights into the Genetic Signatures of Selection and Seed Trait Loci in Cultivated Peanut. J. Adv. Res..

[37] Paysan-Lafosse T., Blum M., Chuguransky S., Grego T., Pinto B.L., Salazar G.A., Bileschi M.L., Bork P., Bridge A., Colwell L. (2023). InterPro in 2022. Nucleic Acids Res..

[38] Arabidopsis Lectin Receptor Kinases LecRK-IX.1 and LecRK-IX.2 Are Functional Analogs in Regulating Phytophthora Resistance and Plant Cell Death. https://apsjournals.apsnet.org/doi/epdf/10.1094/MPMI-02-15-0025-R.

[39] Zhang Z., Xie Q., Jobe T.O., Kau A.R., Wang C., Li Y., Qiu B., Wang Q., Mendoza-Cózatl D.G., Schroeder J.I. (2016). Identification of AtOPT4 as a Plant Glutathione Transporter. Mol. Plant.

[40] Stacey M.G., Osawa H., Patel A., Gassmann W., Stacey G. (2006). Expression Analyses of Arabidopsis Oligopeptide Transporters during Seed Germination, Vegetative Growth and Reproduction. Planta.

[41] Xu P., Tang G., Cui W., Chen G., Ma C.-L., Zhu J., Li P., Shan L., Liu Z., Wan S. (2020). Transcriptional Differences in Peanut (*Arachis hypogaea* L.) Seeds at the Freshly Harvested, After-Ripening and Newly Germinated Seed Stages: Insights into the Regulatory Networks of Seed Dormancy Release and Germination. PLoS ONE.

[42] Zou Z., Chen J., Wu W., Luo J., Long T., Wu Q., Wang Q., Zhen J., Zhao Y., Wang Y. (2023). Detection of Peanut Seed Vigor Based on Hyperspectral Imaging and Chemometrics. Front. Plant Sci..

[43] Müntz K., Belozersky M.A., Dunaevsky Y.E., Schlereth A., Tiedemann J. (2001). Stored Proteinases and the Initiation of Storage Protein Mobilization in Seeds during Germination and Seedling Growth. J. Exp. Bot..

[44] Ellis R.H. (1992). Seed and Seedling Vigour in Relation to Crop Growth and Yield. Plant Growth Regul..

[45] Alqudah A.M., Sallam A., Stephen Baenziger P., Börner A. (2020). GWAS: Fast-Forwarding Gene Identification and Characterization in Temperate Cereals: Lessons from Barley—A Review. J. Adv. Res..

[46] Lever J., Krzywinski M., Altman N. (2017). Principal Component Analysis. Nat. Methods.

[47] Xiao M., Ma Y., Feng Z., Deng Z., Hou S., Shu L., Lu Z. (2018). Rice Blast Recognition Based on Principal Component Analysis and Neural Network. Comput. Electron. Agric..

[48] Wang Z., Huang W., Li J., Liu S., Fan S. (2023). Assessment of Protein Content and Insect Infestation of Maize Seeds Based on On-Line near-Infrared Spectroscopy and Machine Learning. Comput. Electron. Agric..

[49] Fernández-Habas J., Carriere Cañada M., García Moreno A.M., Leal-Murillo J.R., González-Dugo M.P., Abellanas Oar B., Gómez-Giráldez P.J., Fernández-Rebollo P. (2022). Estimating Pasture Quality of Mediterranean Grasslands Using Hyperspectral Narrow Bands from Field Spectroscopy by Random Forest and PLS Regressions. Comput. Electron. Agric..

[50] Purcell S., Neale B., Todd-Brown K., Thomas L., Ferreira M.A., Bender D., Maller J., Sklar P., De Bakker P.I., Daly M.J. (2007). PLINK: A Tool Set for Whole-Genome Association and Population-Based Linkage Analyses. Am. J. Hum. Genet..

[51] Raj A., Stephens M., Pritchard J.K. (2014). fastSTRUCTURE: Variational Inference of Population Structure in Large SNP Data Sets. Genetics.

[52] Bradbury P.J., Zhang Z., Kroon D.E., Casstevens T.M., Ramdoss Y., Buckler E.S. (2007). TASSEL: Software for Association Mapping of Complex Traits in Diverse Samples. Bioinformatics.

[53] Kang H.M., Sul J.H., Service S.K., Zaitlen N.A., Kong S., Freimer N.B., Sabatti C., Eskin E. (2010). Variance Component Model to Account for Sample Structure in Genome-Wide Association Studies. Nat. Genet..

[54] Dash S., Cannon E.K.S., Kalberer S.R., Farmer A.D., Cannon S.B. (2016). PeanutBase and Other Bioinformatic Resources for Peanut. Peanuts.

[55] Quick C., Wen X., Abecasis G., Boehnke M., Kang H.M. (2020). Integrating Comprehensive Functional Annotations to Boost Power and Accuracy in Gene-Based Association Analysis. PLoS Genet..

[56] Eichler E.E., Flint J., Gibson G., Kong A., Leal S.M., Moore J.H., Nadeau J.H. (2010). Missing Heritability and Strategies for Finding the Underlying Causes of Complex Disease. Nat. Rev. Genet..

[57] Li B., Leal S.M. (2008). Methods for Detecting Associations with Rare Variants for Common Diseases: Application to Analysis of Sequence Data. Am. J. Hum. Genet..

[58] Livak K.J., Schmittgen T.D. (2001). Analysis of Relative Gene Expression Data Using Real-Time Quantitative PCR and the 2−ΔΔCT Method. Methods.

