# Supplementary material for: Combining Hyperspectral Techniques and Genome-Wide Association Studies to Predict Peanut Seed Vigor and Explore Associated Genetic Loci
Source: Int J Mol Sci. 2024 Aug 1;25(15):8414. doi: 10.3390/ijms25158414 (PMC11313457; doi:10.3390/ijms25158414)
Supplement: Supplementary file 1 [file ijms-25-08414-s001.zip › ijms-3089581-supplementary.pdf]

Supplementary Materials

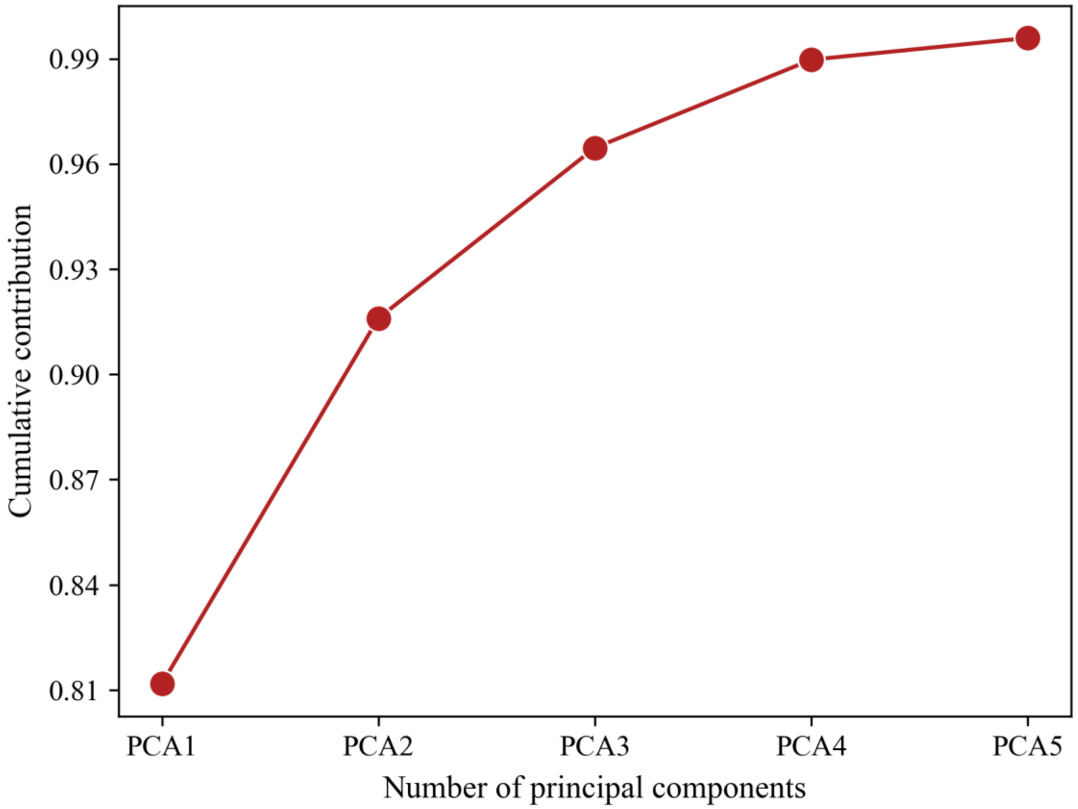

Figure S1. PCA Cumulative contribution.

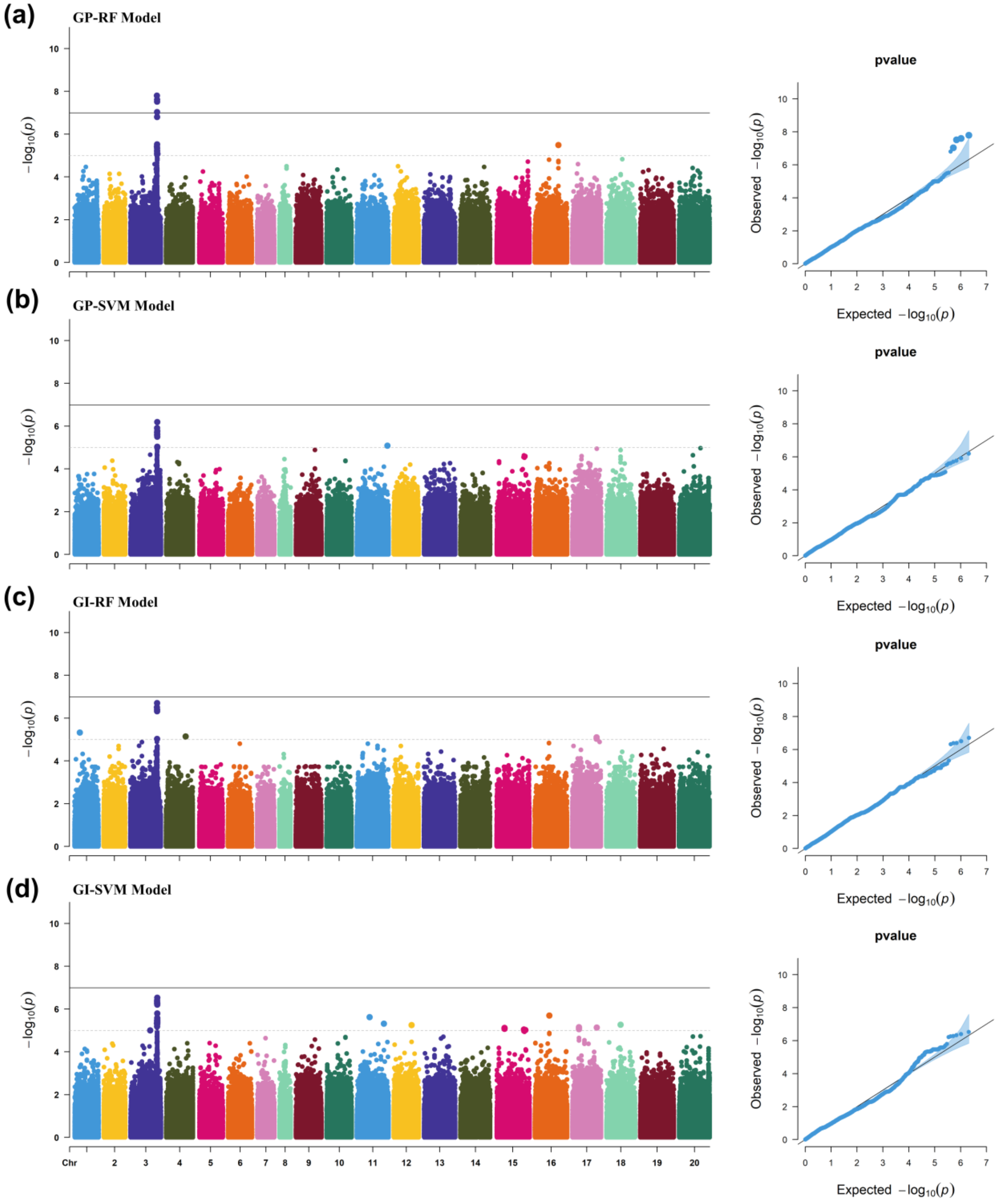

Figure S2. Gene-based association (GBA) results for phenotypic predictive values of different models. (**a**–**d**) GBA Manhattan plots (left) and quantile-quantile plots (right) for phenotypic data predicted by GP-RF, GP-SVM, GI-RF, and GI-SVM, respectively.

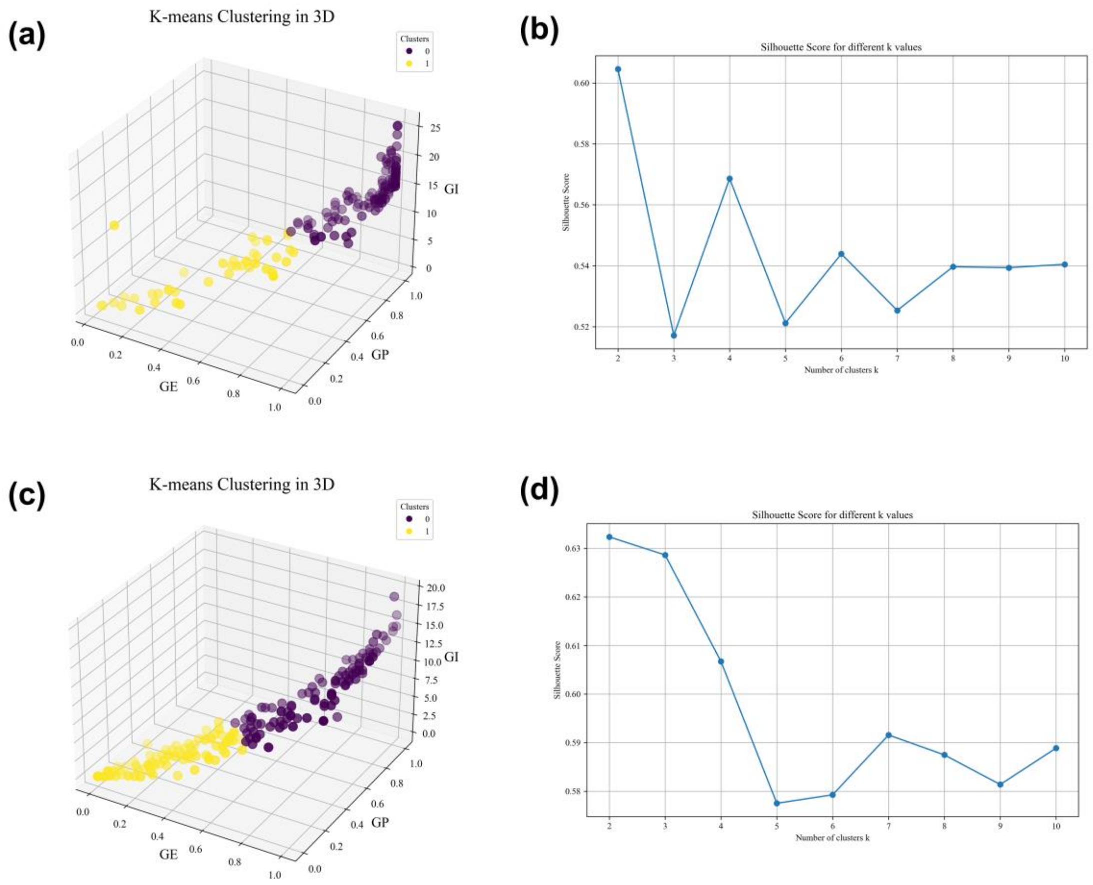

Figure S3. Cluster analysis plot for K-means and Silhouette Score Method plot. (**a,c**) The clustering results for the two batches of materials. (**b,d**) K-value fold plots based on silhouette score.

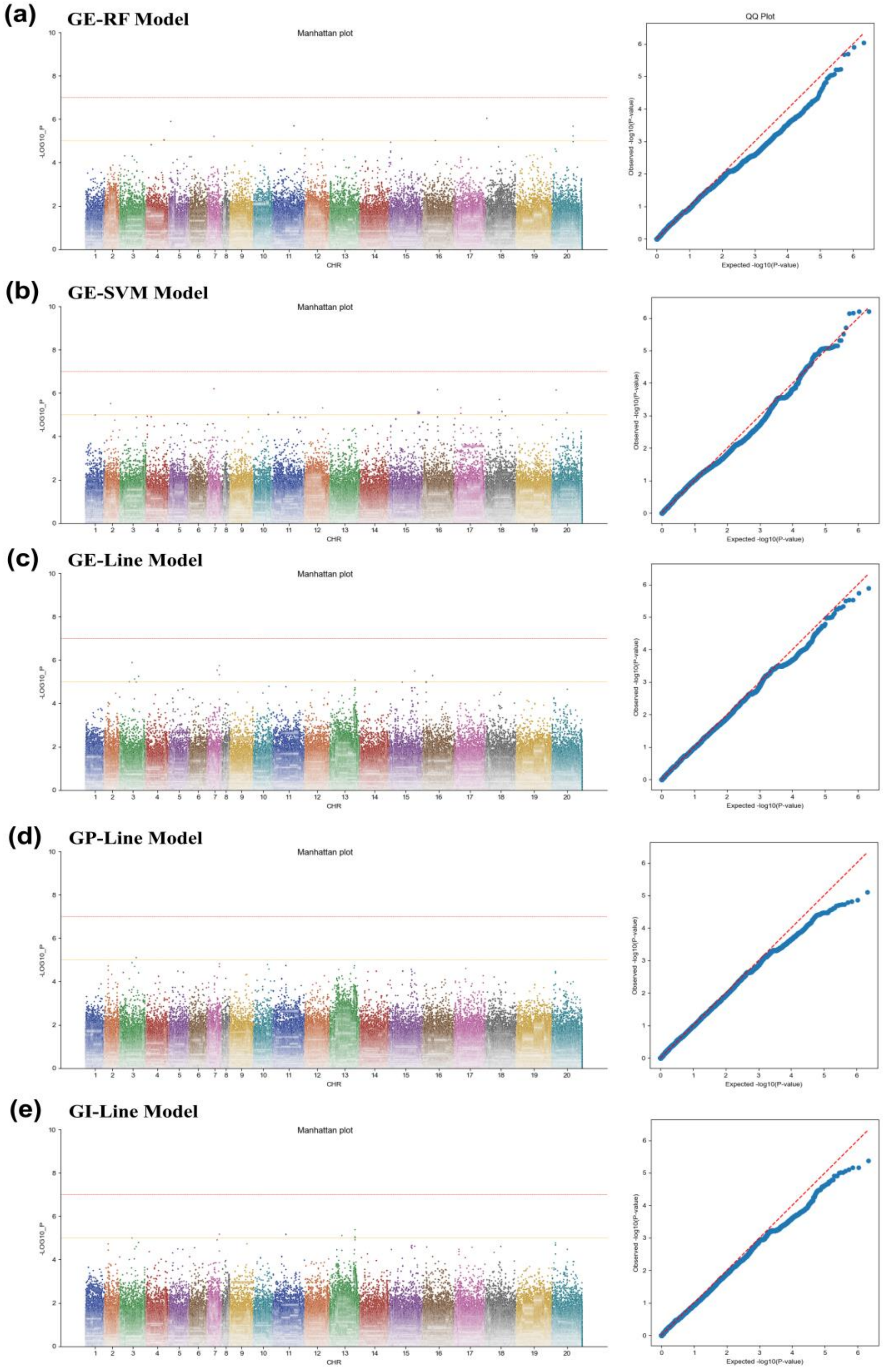
(a)
GE-RF Model
Manhattan plot
QQ Plot
(b)
GE-SVM Model
Manhattan plot
(c)
GE-Line Model
Manhattan plot
(d)
GP-Line Model
Manhattan plot
(e)
GI-Line Model
Manhattan plot
-LOG10_P
CHR
Observed -log10(P-value)
Expected -log10(P-value)

Figure S4. Genome-wide association analysis (GWAS) based on GE and Line-based model prediction data. (**a,b**) GWAS Manhattan plots (left) and quantile-quantile plots (right) for GE predicted by GE-RF, GE-SVM, respectively. (**c**–**e**) GWAS Manhattan plots (left) and quantile-quantile plots (right) for phenotypic data predicted by Line models.

Table S1. Statistics of predictive evaluation metrics for all models.

Trait	Model	R^2	MSE	RMSE
GE	SVM	0.5735	0.0542	0.1819
	RF	0.5219	0.0734	0.2310
	Line	0.5547	0.0586	0.1907
	RT	0.0974	0.1204	0.2673
GP	SVM	0.6247	0.0500	0.1819
	RF	0.5430	0.0583	0.2137
	Line	0.5314	0.0581	0.2411
	RT	0.2364	0.0975	0.3123
GI	SVM	0.6662	11.6315	2.8679
	RF	0.5690	16.2566	3.5628
	Line	0.5130	19.5427	4.4207
	RT	0.1994	30.1681	5.4925

Table S2. Detailed information on genes identified by SNPs.

Genes	CHR	Gene Region	Annotation function
Arahy.7XWF6F	3	[125569785, 125573510]	oligopeptide transporter 4
Arahy.VMLN7L	3	[125514864, 125523060]	protein serine/threonine kinases
Arahy.00RJ5Y	3	[125563665, 125565569]	Protein kinase superfamily protein
Arahy.0811LV	3	[125505612, 125507710]	receptor lectin kinase
Arahy.B3BEZ6	3	[125560407, 125562374]	protein serine/threonine kinases
Arahy.3D4LUS	3	[125585698, 125589622]	animal RPA1 domain protein
Arahy.50LAX6	3	[125566536, 125569030]	protein serine/threonine kinases
Arahy.CLYK6S	3	[125576201, 125577474]	Unknown protein
Arahy.IE46SE	3	[125497757, 125504527]	protein serine/threonine kinases

Table S3. The results of Blastp

Genes	Protein comparison	Identities	E-values
Arahy.VMLN7L	LecRK-IX.1 *(AT5G10530)*	47.09%	0
Arahy.7XWF6F	AtOPT4 *(AT5G64410)*	78.53%	0
